# Serum Diamine Oxidase Levels in Relation to Skin Prick Testing and Specific IgE in Adults with Self-Reported Food Allergy

**DOI:** 10.3390/jcm14175927

**Published:** 2025-08-22

**Authors:** Tomislav Duvančić, Bruno Špiljak, Ivana Ćelap, Nikolina Mandušić, Ivica Lokner, Liborija Lugović-Mihić

**Affiliations:** 1 Department of Dermatovenereology, University Hospital Center Sestre Milosrdnice, 10000 Zagreb, Croatia; md.tduvancic@gmail.com (T.D.); nmandusic@gmail.com (N.M.); 2 University of Zagreb School of Dental Medicine, 10000 Zagreb, Croatia; 3 Department of Oral Medicine, University of Zagreb School of Dental Medicine, 10000 Zagreb, Croatia; bspiljak@sfzg.hr; 4 Department of Laboratory Diagnostics, University Hospital Center Sestre Milosrdnice, 10000 Zagreb, Croatia; ivana.celap@gmail.com; 5 Department of Pulmonology, Special Hospital for Pulmonary Diseases, 10000 Zagreb, Croatia; ivica.lokner@pulmologija.hr; 6 Department of Laboratory Diagnostics, Department of Pulmonology, Special Hospital for Pulmonary Diseases, 10000 Zagreb, Croatia

**Keywords:** food allergy, histamine intolerance, diamine oxidase (DAO), skin prick test, specific IgE, self-reported symptoms, ELISA, adult patients, food hypersensitivity

## Abstract

**Background:** Self-reported food allergy is a common concern among adults and often prompts the use of diagnostic allergy testing. However, serum diamine oxidase (DAO) measurement is rarely performed, despite the fact that symptoms of histamine intolerance can closely mimic those of IgE-mediated food allergy. This study aimed to analyze and compare the results of commonly used food allergy tests in relation to serum DAO levels in this patient population. **Methods:** A total of 61 adult patients with self-reported food-related symptoms were enrolled. All participants underwent skin prick testing and serum DAO measurement using an enzyme-linked immunosorbent assay (ELISA). In addition, serum-specific IgE (sIgE) testing was performed in 29 of the 61 patients. DAO levels were compared across groups based on skin prick testing and sIgE results. **Results:** Fewer than half of the patients had positive test results, 36% for skin prick testing and 38% for sIgE, and 38% showed reduced serum DAO levels. DAO levels did not differ significantly between patients with positive and negative skin prick or sIgE results; however, a slight decrease in DAO was observed in patients with negative sIgE. Patients with negative skin prick tests were significantly older than those with positive results. **Conclusions:** The majority of adults with self-reported food allergies had negative allergy test results and normal DAO levels. Nevertheless, a considerable proportion showed either positive allergy tests or reduced DAO levels, suggesting the potential role of histamine intolerance. These findings underscore the importance of a detailed pre-test clinical assessment that considers both IgE- and non-IgE-mediated mechanisms in patients with suspected food-related hypersensitivity.

## 1. Introduction

Adverse reactions to food represent an etiologically and pathophysiologically heterogeneous group of conditions, generally defined as abnormal responses of an organism to food intake [1,2]. Although the majority of such reactions are non-allergic in nature, patients frequently misclassify them as food allergies. This self-diagnosis may result in a fourfold overestimation of the actual prevalence of true food allergy and often leads to unnecessary dietary restrictions, increasing the risk of nutritional deficiencies [1,3]. It is estimated that up to 20% of the population alters their diet due to suspected adverse reactions to food [1,4,5], while epidemiological studies indicate that true food allergies affect approximately 5% of adults and 8% of children [6,7].

Genuine food allergies may manifest through cutaneous, respiratory, or gastrointestinal symptoms, ranging from mild oral allergy syndrome to potentially life-threatening angioedema [6,8,9]. Diagnostic evaluation typically involves skin prick testing (SPT) and/or measurement of serum-specific IgE (sIgE), which detect allergen sensitization [10,11,12]. While SPT is more sensitive and cost-effective, sIgE testing provides standardized in vitro evaluation and is particularly useful in selected patients [11,12,13,14,15,16]. However, both tests must be interpreted within a clinical context, as sensitization does not always imply clinical reactivity [11,13,17,18,19]. Moreover, the quality and consistency of allergen extracts, along with assay standardization (e.g., ImmunoCAP), remain crucial for diagnostic accuracy [13,14,15]. Despite these advances, more than 20% of individuals with detectable sIgE remain asymptomatic [17,18,19], and neither SPT nor sIgE alone is sufficient to establish a definitive diagnosis [20,21,22].

In recent years, histamine intolerance (HIT) has gained increasing attention as a potential differential diagnosis, particularly in cases where conventional allergy tests are negative but food-related symptoms persist [23,24,25]. Diamine oxidase (DAO) is the primary enzyme responsible for extracellular histamine degradation in the gastrointestinal tract [26]. Reduced DAO activity may lead to histamine accumulation, producing symptoms such as flushing, urticaria, headaches, and gastrointestinal distress—many of which overlap with IgE-mediated reactions [27,28,29,30]. Despite its clinical relevance, DAO is rarely included in routine allergy workups, and interpretation of its serum levels remains a matter of debate.

Given the symptomatic overlap and limitations of existing diagnostic tools, a better understanding of the relationship between DAO status and allergen sensitization is needed. The aim of this study was to investigate the relationship between serum DAO levels and allergen sensitization, determined by SPT and sIgE, in a cohort of adults with self-reported food-related symptoms. Although oral food challenges (OFCs) were not performed, this study aimed to explore diagnostic overlaps and identify possible roles of histamine intolerance in this population.

## 2. Materials and Methods

A total of 61 adult patients who reported various symptoms following food ingestion were enrolled in this study. All patients gave informed consent prior to participation in the study. Due to the observational and exploratory design of the study, randomization and blinding were not applicable. All data were obtained from a predefined cohort of patients consecutively referred for evaluation of suspected food-related symptoms. No control group was used.

Information on suspected trigger foods was collected during clinical interviews, although patients were not always certain about the specific culprit. If symptoms were attributed to histamine-rich foods, these were classified accordingly and included in the subgroup analysis. The reported symptoms included urticaria, pruritus, eczema, dermatitis, oral allergy syndrome, flushing, and angioedema. All participants underwent SPT with a panel of 29 nutritive allergens: cow’s milk, egg, pork, beef, chicken, sardines, beans, soybean flour, wheat flour, gluten, sesame, almond, cocoa, peanut, hazelnut, walnut, strawberry, kiwi, apple, watermelon, orange, banana, pineapple, peach, tomato, tuna, squid, shrimp, and mussels. Skin testing was performed in accordance with established European recommendations for SPT procedures [10]. A standardized panel of 29 nutritive allergens was used for all participants, selected based on prior epidemiological data identifying the most common food allergens in the adult population. All SPTs were performed using commercial allergen extracts (Diater Laboratorio, Spain). A wheal diameter of ≥3 mm was considered a positive result, as per guideline recommendations [10,13]. Prick-by-prick testing with fresh foods was not conducted.

DAO concentrations were measured in venous blood serum samples from all 61 participants using a commercially available enzyme-linked immunosorbent assay (ELISA) kit (IDK DAO ELISA, Immundiagnostik AG, Bensheim, Germany; lot valid from 2015-07-22), following the manufacturer’s instructions. Only DAO concentration was measured; enzymatic activity was not assessed due to test limitations and unavailability of validated activity-based assays in the routine laboratory setting. According to the manufacturer’s guidelines, DAO levels were classified as follows: <3 U/mL—distinctly decreased; 3–10 U/mL—slightly decreased; ≥10 U/mL—normal.

In addition, sIgE antibody testing was conducted in a subgroup of 29 patients using the ImmunoCAP system (Thermo Fisher Scientific, Waltham, Massachusetts, United States). This subgroup was defined based solely on the availability of sIgE results in patient records. A total of 20 individual food allergens were tested using whole allergen extracts, not molecular components. Multiplex platforms such as ISAC were not used. The panel included the following food allergens: soybean flour, wheat flour, gluten, sesame, almond, hazelnut, walnut, strawberry, kiwi, apple, orange, banana, pineapple, peach, tomato, tuna, squid, shrimp, mussels, and melon. sIgE measurement was performed in accordance with standardized procedures for in vitro allergen-specific IgE testing [13]. A concentration of ≥0.35 kU/L was used as the positivity threshold for specific IgE.

Participants ranged in age from 20 to 79 years (median: 47 years; interquartile range: 39–60), and 80% were female. Both SPT and sIgE results were considered positive if at least one allergen yielded a positive finding in the respective test.

Statistical analysis was performed using standard non-parametric and regression methods. Normality of data distribution was assessed using the Kolmogorov–Smirnov and Shapiro–Wilk tests. As the data did not meet the criteria for normal distribution, Spearman’s correlation was used to evaluate relationships between continuous variables. Differences in DAO levels between sexes were assessed using the Mann–Whitney U test, while the Kruskal–Wallis test was used for differences between sex–age groups. Fisher’s exact test and the χ^2^ test were used for comparisons of proportions. Multiple linear regression was applied to identify potential predictors of DAO levels, including age, gender, SPT result, and sIgE result. Predictors of histamine intolerance—defined as DAO levels <10 U/mL—were assessed using multiple logistic regression analysis. All statistical analyses were performed using IBM SPSS Statistics version 22 (IBM Corp., Armonk, NY, USA). Statistical significance was set at *p* < 0.05.

SPT interpretation and allergen standardization were performed according to established guidelines involving food allergens in adult populations [10,13].

## 3. Results

### 3.1. Skin Prick Test (SPT), Specific IgE (sIgE), and Age Differences

**Figure 1:**
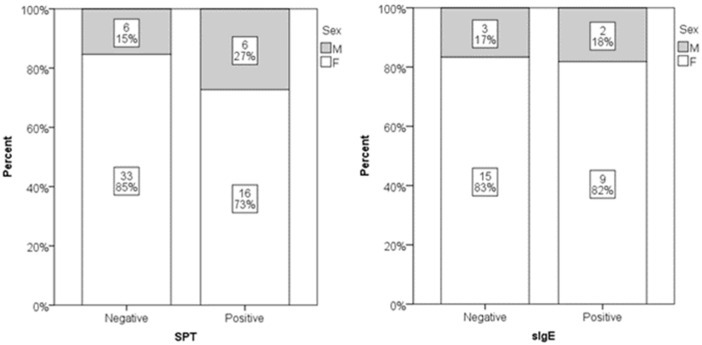
Distribution of genders in positive and negative SPT (*p* = 0.586) and sIgE subjects (*p* = 0.228). Relative numbers and percentages are presented.

**Figure 2:**
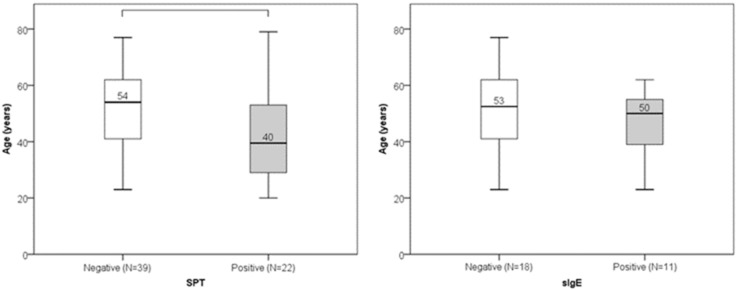
Age distribution in positive and negative SPT groups (*p* = 0.008) and sIgE groups (*p* = 0.334). Median value of age is presented numerically.

Out of 61 participants, 22 (36%) had a positive SPT result, while sIgE was positive in 11 out of 29 tested subjects (38%). No statistically significant gender differences were found in SPT or sIgE positivity (Figure 1). Age did not significantly differ between individuals with positive and negative sIgE results. However, individuals with negative SPT results were significantly older than those with positive SPT results (median age 54 vs. 40 years; *p* = 0.008) (Figure 2). Low serum DAO levels (defined as <10 U/mL), suggestive of possible histamine intolerance, were detected in 23 out of 61 subjects (38%). When compared to a hypothetical prevalence of 50% in the general population, this difference was not statistically significant.

### 3.2. DAO Levels in Relation to Age and Gender

**Figure 3:**
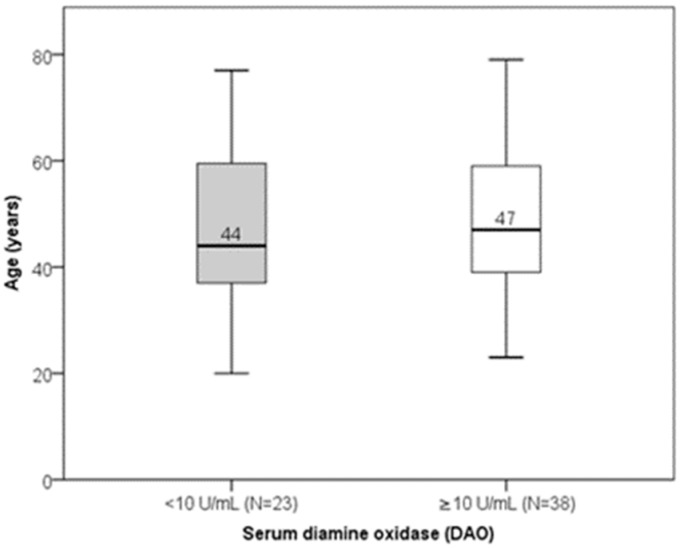
Distribution of age according to DAO levels (*p* = 0.806). Median value of age is presented numerically.

**Figure 4:**
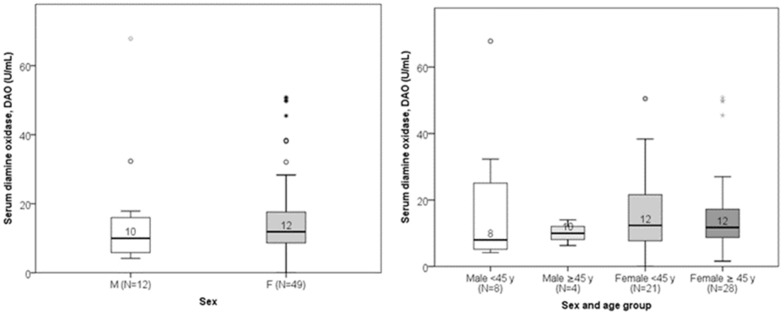
Comparison of DAO levels between males and females (*p* = 0.238) and sex–age combination (*p* = 0.695). The median value of DAO is presented numerically.

Serum DAO concentrations ranged from 0.02 to 67.80 U/mL (median: 11.77 U/mL; interquartile range: 7.84–11.71 U/mL). There was no significant linear correlation between DAO and age (Spearman’s r = 0.035; *p* = 0.789). Age did not differ significantly between subjects with low versus normal DAO levels (Figure 3). DAO levels also did not significantly differ between genders or sex–age combination groups (Figure 4). Low DAO levels were found in 6 of 12 males (50%) and in 17 of 49 females (35%); however, this difference was not statistically significant.

### 3.3. DAO Levels in Relation to SPT and sIgE Results

**Figure 5:**
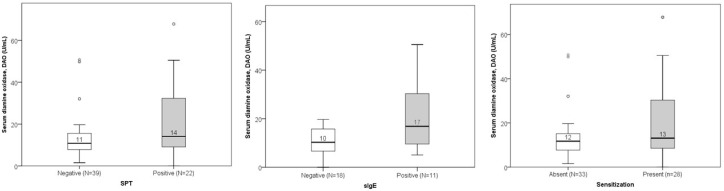
Comparison of DAO levels between non-sensitized and sensitized subjects according to the results of SPT (*p* = 0.167), sIgE (*p* = 0.065), and combined tests (patients with either positive SPT or positive sIgE; *p* = 0.238). The median value of DAO is presented numerically.

DAO levels did not significantly differ between groups with positive versus negative SPT or sIgE results. However, a slight (non-significant) increase in median DAO concentration was observed in patients with positive sIgE results (Figure 5).

**Figure 6:**
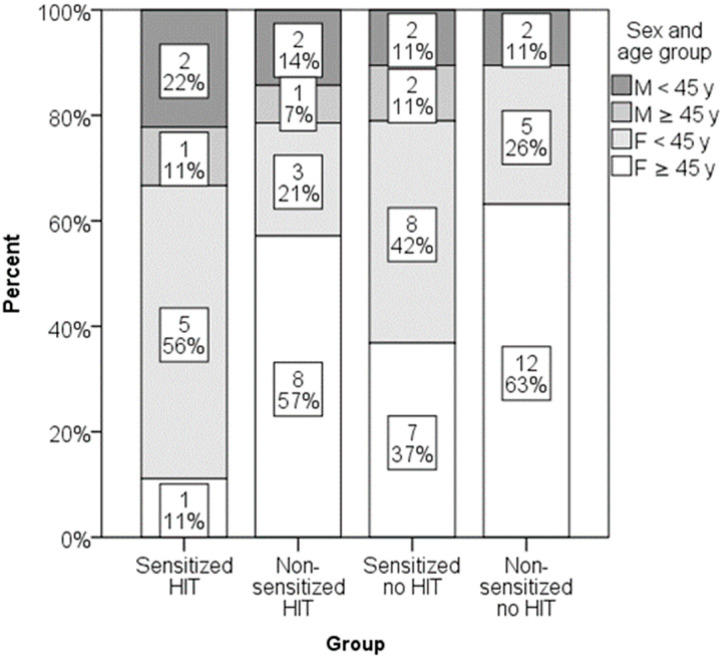
Distribution of sex and age groups in groups formed by the presence of sensitization and histamine intolerance (*p* = 0.380).

Low DAO levels were found in 16 out of 39 participants (41%) with negative SPT results and 7 out of 22 participants (32%) with positive SPT results. The difference was not statistically significant. Similarly, low DAO levels were found in 9 out of 18 participants (50%) with negative sIgE and 3 out of 11 participants (27%) with positive sIgE. Again, this difference was not statistically significant. When patients with either positive SPT or positive sIgE were merged in one group of sensitized patients and DAO levels were compared, no significant differences were found (Figure 5). Totals of 32% (9/28) of sensitized patients and 42% (14/33) of non-sensitized patients had histamine intolerance. Sex–age distribution in groups is presented in Figure 6. Some groups had a low number of participants, and differences did not reach the level of statistical significance.

Among foods identified through SPTs, the most frequently detected allergens were tomato (n = 6), beans (n = 5), almond (n = 5), hazelnut (n = 5), apple (n = 5), orange (n = 5), and peach (n = 5). In sIgE analyses, tomato (n = 6), hazelnut (n = 5), peach (n = 4), and shrimp (n = 4) were the most commonly identified allergens. Only three participants had concordant positive results for the same food in both SPT and sIgE testing (banana and tomato in one patient, pineapple in another, and apple in the third). Patients reported a variety of food-related triggers during clinical interviews; however, many were unsure about the specific food responsible for their symptoms. Therefore, a targeted analysis was conducted on histamine-rich food consumption across subgroups. The highest frequency of histamine-rich food consumption was observed in sensitized patients with histamine intolerance, defined by a DAO level < 10 U/mL. Nevertheless, this difference did not reach statistical significance (Table 1).

Multiple linear regression analyses, controlling for age, gender, SPT results, and sIgE, did not identify any significant predictors of DAO levels. Likewise, logistic regression models evaluating predictors of histamine intolerance (DAO < 10 U/mL) were not statistically significant.

## 4. Discussion

Adverse reactions related to food ingestion are highly variable and may involve multiple organ systems or mechanisms [1,31,32,33,34]. While IgE-mediated allergies represent a well-characterized subset, other immune and non-immune mechanisms also contribute to clinical symptoms [2,35,36,37]. Onyimba et al. [5] categorized food-related symptoms by affected systems—gastrointestinal (e.g., abdominal pain, diarrhea), skin (e.g., urticaria, pruritus), respiratory (e.g., wheezing, nasal congestion), and systemic (e.g., hypotension, fatigue)—but only about half of such adverse reactions are attributable to IgE-mediated mechanisms. Consequently, self-reported food allergy often leads to an overestimation of true prevalence, sometimes fourfold higher than confirmed cases, and results in unnecessary dietary restrictions and nutritional risks [1,3].

In the adult population, food intolerances such as lactose, fructose, and wheat intolerance are significantly more prevalent than food allergies [38,39,40]. Differential diagnoses such as irritable bowel syndrome and intestinal dysbiosis further complicate clinical evaluation [40,41,42]. Recent meta-analyses reveal that self-reported food allergy affects up to 20% of Europeans during their lifetime, though objective testing confirms a much lower prevalence, approximately 6–8% [43,44]. The OFC remains the diagnostic gold standard, but its practical limitations (cost, risk of anaphylaxis) prompt the use of surrogate tests like SPT and sIgE assays [45,46,47].

While these tests are less invasive, they vary in diagnostic accuracy. For example, a recent systematic review showed that SPT and sIgE testing demonstrate high sensitivity and specificity for peanut and tree nut allergies but are less reliable for allergens like sesame, soy, wheat, and shrimp [48,49]. Sensitivity ensures that a negative test likely rules out the condition, while specificity confirms a diagnosis when the result is positive [14,49,50].

Another condition mimicking food allergy is HIT, caused by reduced activity of DAO, the main enzyme responsible for degrading dietary histamine [24,26,28]. Symptoms may include gastrointestinal (e.g., bloating, diarrhea) and extraintestinal manifestations such as flushing, urticaria, headache, or hypotension [22,23,27,28].

In our study cohort, 36% of participants had positive SPT results and 38% had positive sIgE results, with no statistically significant gender differences observed. Age, however, significantly differed between those with positive and negative SPT results, with older individuals being more likely to have negative tests (*p* = 0.008). This cohort consisted predominantly of female patients, many of whom experienced chronic or recurrent symptoms. Notably, the presence of sensitization did not correlate with increased DAO levels or with the occurrence of symptoms.

Consistently with previous findings [51,52], it is important to state that some conditions, such as perioral inflammatory conditions (e.g., cheilitis and perioral dermatitis), are frequently multifactorial in origin, influenced by behavioral habits, atopic predisposition, environmental exposures, impairment of the epidermal barrier, and psychological stress. The complex interplay of these factors may obscure the clinical picture, making allergic testing both a useful tool and a potential confounder in the diagnostic process.

Measurement of serum DAO levels via enzyme-linked immunosorbent assay (ELISA) is a frequently used diagnostic tool. Common reference cutoffs classify <3 U/mL as markedly decreased, 3–10 U/mL as slightly reduced, and >10 U/mL as normal [30]. In a recent population-based study, nearly half of participants exhibited DAO levels below normal [30]. In our cohort, 38% of patients had DAO < 10 U/mL, with no significant correlation between DAO and age, gender, or SPT and sIgE status.

It should be noted that only DAO concentration was measured, not enzymatic activity. Although both parameters are relevant, activity-based assays are less widely standardized and were not available at the time of testing. This may limit the interpretation of DAO levels in terms of their actual biological functionality.

Specifically, 41% of SPT-negative patients and 32% of SPT-positive patients had low DAO levels, while 50% of sIgE-negative and 27% of sIgE-positive patients exhibited low DAO. None of these differences reached statistical significance. Regression models including age, gender, and allergy test results failed to identify significant predictors of DAO level. Interestingly, the proportion of patients with low DAO levels was similar to those with positive SPT and sIgE results. This underscores the importance of considering histamine intolerance in patients with allergy-like symptoms but negative allergy tests.

In line with previous studies [53,54], we found that the majority of patients with self-reported food allergy had negative objective tests and normal DAO values. Nevertheless, a considerable subset exhibited either sensitization or low DAO levels, suggesting that non-IgE-mediated mechanisms—particularly histamine intolerance—warrant clinical attention. Furthermore, the observation that low DAO levels were also present in both sensitized and non-sensitized individuals reinforces the conclusion that neither SPT, sIgE, nor DAO alone is adequate for diagnosis.

Additionally, patients with both sensitization and low DAO showed the highest—but still non-significant—rates of histamine-rich food consumption and symptoms, suggesting that overlapping mechanisms may contribute to clinical presentation in this subgroup. This is further supported by the regression analysis, which failed to identify any significant predictors of low DAO.

Before initiating extensive diagnostic testing or recommending dietary restrictions, both IgE-mediated allergy and other mechanisms such as histamine intolerance should be considered in patients presenting with food-related complaints. Moreover, a recent study by Krišto et al. [55] has also emphasized the interplay between gut microbiota and immune-mediated conditions such as chronic spontaneous urticaria, suggesting that alterations in the microbiome might modulate histamine-related pathways and inflammatory responses.

Several limitations of this study should be acknowledged. First, the absence of a gold-standard diagnostic test such as the OFC restricts the ability to definitively confirm or exclude food allergy or intolerance. Second, the modest sample size and subgroup stratification may have reduced the power to detect significant associations. Third, the lack of randomization and potential for selection bias due to the observational design should be considered when interpreting the results. Additionally, only serum DAO concentrations were measured in this study, without assessment of enzymatic activity. Although concentration assays are commonly used due to their availability and standardization, they do not necessarily reflect functional activity of DAO in vivo. The lack of DAO activity measurements represents a methodological limitation, as reduced enzyme activity—not just lower concentration—may be more relevant in explaining histamine intolerance symptoms. Future studies should consider incorporating activity-based assays for a more accurate clinical interpretation. Finally, dietary intake of histamine-rich foods was assessed retrospectively and not standardized across participants, which limits the strength of inferences regarding its relationship with DAO levels and symptoms.

## 5. Conclusions

In this study of adults with self-reported food-related symptoms, 36% of participants had positive skin prick test results and 38% had positive specific IgE, while 38% showed reduced serum diamine oxidase (DAO) levels (<10 U/mL). Despite the absence of statistically significant associations between these markers, a considerable subset of patients demonstrated either IgE-mediated sensitization or possible histamine intolerance.

These findings underscore the importance of a thorough clinical evaluation prior to initiating allergy testing or dietary modifications. Neither skin prick testing, specific IgE measurement, nor DAO activity alone proved sufficient to explain symptoms across the entire cohort, highlighting the complexity of food-related adverse reactions. Particularly in cases with negative allergy test results, non-IgE-mediated mechanisms such as histamine intolerance should be considered.

Future studies are warranted to validate the clinical utility of serum DAO testing, define standardized cut-off values, and explore the diagnostic value of combining SPT, sIgE, and DAO assessments. Developing integrated diagnostic algorithms may help distinguish between true food allergy and histamine intolerance more reliably in clinical practice.

## Figures and Tables

**Table 1:** Comparison of use of histamine-rich food and presence of symptoms in groups formed by presence/absence of sensitization and histamine intolerance (HIT).

Variable		Sensitized HIT (N = 9)	Non-Sensitized HIT (N = 14)	Sensitized No HIT (N = 19)	Non-Sensitized No HIT (N = 19)	*p* *
Histamine-rich food	No	2 (22%)	8 (57%)	11 (58%)	8 (42%)	
	Yes	7 (78%)	6 (43%)	8 (42%)	11 (58%)	0.276
Symptom	Absent	1 (11%)	0 (0%)	0 (0%)	2 (11%)	
	Present	8 (89%)	14 (100%)	19 (100%)	17 (90%)	0.293

* χ^2^ test.

## Data Availability

Data are available upon request to the corresponding author.
